# Conformational determinants necessary for secretion of *Paecilomyces thermophila* β-1,4-xylosidase that lacks a signal peptide

**DOI:** 10.1186/s13568-018-0542-2

**Published:** 2018-01-24

**Authors:** Yalin Yang, Juan Li, Qiang Yu, Junxiu Hou, Chenchen Gao, Dong Li, Yang Liu, Chao Ran, Zhigang Zhou

**Affiliations:** The Key Laboratory for Feed Biotechnology of the Ministry of Agriculture, Feed Research Institute, Chinese Academy of Agricultural Sciences, Beijing, 100081 People’s Republic of China

**Keywords:** Non-classical secretion, Secretory conformational determinants, Blades, *Escherichia coli*

## Abstract

In this study, we investigated the secretion mechanism of the hyper-secretion signal peptide-lacking β-xylosidase PtXyl43, a non-classically secreted protein, from the fungus *Paecilomyces thermophila* in *Escherichia coli* BL21(DE3). PtXyl43 secretion is a two-step process, and the second step is accompanied by cell periplasmic leakage, indicating that PtXyl43 secretion is the result of semi-specific secretion. Homology modeling of PtXyl43 suggested that PtXyl43 had a canonical GH43 family β-xylosidase structure containing five blades. Seventeen blade deletions or circular mutants were designed to identify the conformational motif(s) involved in secretion. These mutants were expressed as recombinant, codon-optimized proteins in *E. coli*. Notably, only mutants containing blades 2–4 were effectively secreted. Blades 2–4 are necessary for secretion, but it appears that blade 1 or 5 must be present to maintain the structure of blades 2–4. Simultaneous deletion of blades 1 and 5 dramatically reduces excretion. The covalent and sequential linking of blades of 2, 3 and 4 are important for the excretion of mutants, as separate blades of 2 and 3 or 3 and 4 abolishes excretion. Fusion with PtXyl43 promotes the excretion of GFP from the periplasm to the extracellular milieu, which suggested that PtXyl43 had the potential to carry proteins. This study provides new insights into secretory mechanism of secretable signal peptide-lacking proteins in *E. coli*. To our knowledge, this is the first to definitively identify the conformational determinants for secretion of a signal peptide-lacking GH43 family β-xylosidase. This finding also has application potential for the secretion of recombinant proteins.

**Electronic supplementary material:**

The online version of this article (10.1186/s13568-018-0542-2) contains supplementary material, which is available to authorized users.

## Introduction

*Escherichia coli* is one of the most widely used hosts for the recombinant proteins expression in laboratory research and industrial production. However, laboratory strains of *E. coli* often do not secrete large quantities of proteins compared with other expression hosts because of the two membranes feature specific to Gram-negative bacteria and its insufficient capacity of the transport machinery (Low et al. 2013). A few of remarkable yields for secreted proteins are only obtained through high-density fermentation using fine-tuning expression systems (Georgiou and Segatori 2005). Recently, the GH family 43 β-xylosidase PtXyl43 from the fungus *Paecilomyces thermophila* was found to be secreted from *E. coli* in good yield (Teng et al. 2011). Interestingly, PtXyl43 does not encode a signal peptide (Teng et al. 2011) and belongs to non-classically secreted protein. Despite the many reports about extracellular secretion of signal peptide-lacking proteins in various bacteria, there is no conclusive and universally applicable pathway identified so far. The most widespread opinion is that excretion of signal peptide-lacking proteins is due to cell lysis. However, there are growing evidences and hints that contradict cell lysis as a primary secretion mechanism for signal peptide-lacking proteins (Wang et al. 2013; Gotz et al. 2015; Ebner et al. 2016). Several potential secretion pathways, such as SecA2-dependent export pathway (Gram-positive bacteria), major autolysin-related pathway (Gram-positive bacteria), type III secretion system (T3SS, Gram-negative bacteria) (Wang et al. 2013), were found to export a small proportion of non-classical proteins in the specific bacteria. However, the secretion mechanisms of most non-classical proteins are still not clear. A greater understanding of non-classical protein secretion has application significance in therapeutic purposes or protein production.

More and more research supports the idea of a conformational signal for recognition by the secretion machinery (Filloux 2010). However, common characteristics of the secretory conformation motif have not been identified. Based on the locations, secretory target motifs were classified into three groups. Some of secretory target motifs are located at the N- or C-terminus of the proteins. For example, the folding of a C-terminal passenger domain segment of the *E. coli* autotransporter EspP is required for the passenger domain secretion (Peterson et al. 2010). The integrity of loop 3 of the N-terminal Fn3 domain seems to be important for a type II secretion system (T2SS) protein PelI secretion (Pineau et al. 2014). Some secretory conformation motifs are involved non-C or N-terminal regions. Five sequential α-helices in the central domain II of exotoxin A serve as the conformational motif necessary for secretion via the type II secretory system (Voulhoux et al. 2000). Some secretory conformation determinants involve multiple regions throughout the protein. For example, multiple regions throughout the *Vibrio cholerae* TcpF influence extracellular secretion by the type IV toxin-coregulated pilus (TCP) (Krebs et al. 2009). The importance of the conformation in the non-classical protein secretion was also found in bacteria. The retentions of N-terminal seventh amino acid and the C-terminal third amino acid are important for the quenching enzyme AIO6 secretion in *Bacillus subtilis* (Pan et al. 2016). The C- and N-terminus, and two hydrophobic domains, are required for structural stability and non-classical secretion of a typical cytoplasmic protein d-psicose 3-epimerase (Zhao et al. 2017). The structure/secretion relationships of the non-classically secreted proteins will help to better understand the protein recognition mechanisms of non-classical secretion. However, little is known about the structure/secretion relationships of the non-classically secreted proteins in *E. coli*, especially laboratory *E. coli*.

β-Xylosidases, key enzymes for xylan degradation, have great potential for use in many biotechnological applications, especially for the food, bio-conversion, and pulp and paper industries (Sunna and Antranikian 1997). In this study, we addressed the hyper-secretion mechanism of signal peptide-lacking β-xylosidase PtXyl43 in *E. coli* and further assessed the conformational requirements for PtXyl43 secretion using PtXyl43 blade-truncated or circular mutants. The ability of PtXyl43 as a carrier to export proteins was also investigated.

## Materials and methods

### Bacterial strains and culture conditions

All plasmids and bacterial strains used in this study are shown in Additional file 1: Table S1. *E. coli* Trans1-T1 (Transgen, Beijing, China) was used for cloning procedures. *E. coli* BL21 (DE3) (Novagen, Beijing, China) was used for expression of the recombinant proteins. Cells were incubated in Luria-Bertani (LB) broth (100 μg/mL ampicillin or 50 µg/mL kanamycin) at 37 °C.

### Molecular biology techniques and plasmid construction

Restriction enzymes were obtained from Fermentas (Beijing, China) or New England Biolabs (Beijing, China). DNA polymerase LA Taq and T4 DNA ligase were obtained from Takara (Beijing, China). TIANpure Mini Plasmid and Gel purification kits were from Tiangen (Beijing, China). All enzymes and kit reagents were used according to the manufactures’ instructions. The primers used in this study are summarized in Additional file 1: Table S2.

Codon usage in X12345 for expression in *E. coli* (http://www.kazusa.or.jp/codon/) was optimized using the codon optimization tool JCat (http://www.jcat.de/) and the optimized codon gene was synthesized by Shanghai Generay Biotech Co., Ltd. (Shanghai, China). The full-length codon-optimized PtXyl43 (denoted X12345 herein) was inserted into the *Xba*I and *Xho*I sites of pET-28(a) to generate the vector pETX12345.

We also designed nucleotide sequences encoding the blade deletion or circular X12345 mutants as shown in Fig. 2b. The mutant genes were amplified by conventional PCR techniques or by partially overlapping primer-based PCR (Tao and He 2004) and individually cloned into the *Nco*I/*Xho*I sites of a pET-28(a) vector to construct the plasmids as listed in Additional file 1: Table S1.

To prepare plasmids encoding the passenger-carrier constructs, DNA sequences containing in the following order from the 5′ to 3′ end: the *Nco*I cleavage site, X12345 without a stop codon, the nucleotide sequence encoding the flexible (GGGGS)_3_ linker, the nucleotide sequence encoding the TEV-protease cleavage site (ENLYFQG), and the *Eco*RI recognition site, were constructed and PCR amplified (with X12345 acting as the carrier in the final construct). After digestion with the corresponding restriction endonucleases (NEB or Fermentas, Beijing, China), segment was ligated into pET-28a(+) to construct the carrier plasmid pX12345LT. The GFP, AIO6, and chitinase genes from the plasmids pEGFP-N1 (BD Biosciences Clontech, Palo Alto, CA, USA), pET-28a-aiio-AIO6 (used as passenger plasmid in this study) (Zhang et al. 2011), and pCHIX (used as passenger plasmid in this study) (Xu et al. 2016), respectively, and the hIL-2 gene which synthesized by Shanghai Generay Biotech Co., Ltd. (Shanghai, China), were PCR amplified. All these resulting PCR products, none of which contained a stop codon, were introduced into *Eco*RI site immediately upstream of their 5′-terminus and *Not*I site immediately downstream of their 3′-terminus. After digestion with *Eco*RI and *Not*I (NEB or Fermentas, Beijing, China), these genes were each individually ligated into the carrier plasmid containing X12345 digested with the same restriction enzymes to construct the passenger-carrier plasmids pX12345–GFP, pX12345–CHIX, pX12345–AIO6, and pX12345–hIL2. GFP and hIL-2 genes also were ligated into pET28a(+) which was digested with the same enzymes to construct passenger plasmids pGFP and phIL2.

All plasmids were verified by sequencing, and then the confirmed plasmids separately were transformed into *E. coli* Trans1-T1 and BL21 (DE3) competent cell samples.

### Protein expression

For expression of each recombinant protein, first, *E. coli* BL21 (DE3) samples each transformed with one of the expression plasmids individually were cultured in 50 mL LB broth with shaking at 200 rpm for ~ 14 h at 37 °C for use as a seed culture. Each culture was then diluted 1:100 with Terrific Broth (TB) medium, 50 μg/mL kanamycin, pH 6.4 and cultured with shaking at 200 rpm at 37 °C until the OD_600_ of the culture was 0.8. Then lactose was added [final concentration, 2% (w/v)] to induce expression of the target protein, and each culture was incubated for 0–48 h at 20 or 33 °C. Each cell pellet and culture supernatant were separated by centrifugation at 10,000×*g* for 10 min at 22 °C to avoid cold shock. Proteins in the supernatants were precipitated with two volume of ice-cold acetone, and then the acetone precipitation and cell pellets were subjected to SDS-PAGE (12% (w/v) acrylamide) and western blotting analysis. Culture growth was monitored by measuring optical density at 600 nm with the BioPhotometer plus of Eppendorf AG (Hamburg, Germany).

### Subcellular fractionation

Osmotic shock was used to isolate the *E. coli* periplasm from the cytoplasm proteins after expression of the constructs (Neu and Heppel 1965). Briefly, recombinant cultures (0.8 mL) at various induction time points were taken and centrifuged to separate into medium (medium fraction, M) and cell pellets (cellular fraction, C). Cell pellets were resuspended thoroughly in 0.6 mL of hypertonic solution (30 mM Tris–HCl (pH 8.0), 20% sucrose and 1 mM EDTA) and shaked slowly at room temperature for 10 min. Following centrifugation at 10,000×*g* at 4 °C for 10 min, supernatants were saved (periplasmic fraction, P1), and pellet were resuspended in 0.6 mL of ice-cold 5 mM MgSO_4_ (hypotonic solution) and shaked slowly for 10 min on ice. After centrifugation at 10,000×*g* at 4 °C for 10 min, the shocked cells (spheroplasts faction, S) and the supernatants (periplasmic fraction, P2) were saved. To isolate the soluble and insoluble fractions, cell pellets (cell fraction, C) from 0.8 mL of culture were resuspended by 0.5 mL of BugBuster Protein Extraction Reagent and incubated on a shaking platform for 10–20 min at room temperature. After centrifugation at 4 °C at 13,000×*g* for 20 min, pellets (the insoluble fraction, IF) and supernatants (the soluble fraction, SF) were saved. All the supernatants, such as medium fraction, periplasmic fraction (P1 and P2) and soluble fraction, were mixed with equal volume of acetone, respectively, then placed on − 80 °C for 1 h followed by centrifugation at 13,000×*g* for 10 min, and the corresponding resulting pellets were saved. To ensure comparability between different fractions, the separated fractions pellets were resuspended in the same volume of PBS buffer as that was used to suspend the cell pellet (cellular fraction, C) with OD_600_ of 10. Then 5× SDS-PAGE sample loading buffer was added to each fraction, and the fractions were boiled at 100 °C for 10 min and centrifuged at 4 °C at 13,000×*g* for 10 min before loading on gels for SDS-PAGE and western blotting. The same volume of each supernatant was loaded per lane.

### SDS-PAGE and western blotting

Proteins in the culture medium, a sucrose hypertonic solution, the periplasm were isolated by precipitation with two volumes of ice-cold acetone. Protein solution was mixed with equal volume of acetone and kept at − 70 °C for 30 min, − 20 °C for 1 h, and then centrifuged at 4 °C at 13,000×*g* for 15 min. The pellets were air dried for at least 1 h and then suspended in PBS buffer (7.4). After electrophoresis loading buffer was added, all samples were boiled at 100 °C for 5 min and then centrifuged at 13,000×*g* for 10 min, and 10 μL of each loaded onto a SDS-PAGE gel (TGX Stain-Free™ FastCast™ Acrylamide Kit, 12%, Bio-Rad, Cat. No. 161-0185). After electrophoresis, the proteins were transferred to Immobilon^®^-PSQ membrane (Millipore, 0.2 μm) for immunoblotting. To detect the His-tag, a mouse monoclonal anti-His antibody (Tiangen, Beijing, China) was used. GroEL was detected using a rabbit polyclonal antiserum against GroEL (Sigma, Shanghai, China). Monoclonal antibody against MBP was from Beijing Protein Innovation Co., Ltd (Beijing, China).

### Mass spectrometry (MS)

The culture supernatant from *E. coli* BL21(DE3) that expressed X12345 for 48 h after lactose induction was loaded onto a pre-equilibrated Ni^2+^-NTA column and washed with 4 column volumes of elution buffer (pH 8.0) containing 20, 40, 60, 80, or 200 mM imidazole in 300 mM NaCl, 50 mM NaH_2_PO_4_ solution (flow rate: 1 mL/min). The protein fraction that was eluted with 60 mM imidazole was subjected to quadrupole time-of-flight (Q-TOF) MS at the Laboratory of Proteomics, Institute of Biophysics, Chinese Academy of Sciences, Beijing, China.

### Field emission scanning electron microscopy (FESEM)

After lactose induction for 8, 12 or 24, *E. coli* BL21(DE3)/pX12345 or BL21(DE3)/pET28a cells were harvested by centrifugation (2200×*g*, 5 min), and washed twice with PBS buffer (pH 7.0). Samples were fixed with 2.5% glutaraldehyde overnight at 4 °C, then washed three times with 0.1 M PBS (pH 7.2) for 10 min each, dehydrated through gradient ethanol elution [30, 50, 70, 85 and 95% (v/v)] for 15 min at each concentration, and followed by 100% (v/v) ethanol three times for 15 min each. Samples were critically point dried in a CO_2_ atmosphere (BAL-TEC CPD 030, Leica Microsystems GmbH, Wetzlar, Germany), sputter-coated with gold palladium by Hitachi ion sputter (E-1045, Hitachi Co., Tokyo, Japan), then observed with a Hitachi SU8010 FESEM (Hitachi Co., Tokyo, Japan) at the Public technology service center, Institute of Microbiology, Chinese Academy of Sciences, Beijing, China.

### Membrane sensitivity

Susceptibility testing was performed by the broth micro dilution method (CLSI 2012) with slight modification for erythromycin, rifampicin, deoxycholate or lysozyme. After lactose induction for 8, 12 or 24 h, *E. coli* BL21(DE3)/pX12345 or BL21(DE3)/pET28a cells were harvested by centrifuge for 5 min at 2200×*g*, then diluted to 1 × 10^5^ colony forming units (CFU)/mL in LB broth. Aliquots of 100 μL of cells (~ 1×10^5^ CFU/mL) were incubated with serial twofold dilutions of each test compound on 96-well plates at 35 °C in ambient air. Growth was measured spectrophotometrically (VersaMax Microplate Reader, Molecular Devices, Sunnyvale, CA, USA) as OD_600_ at ~ 16 h incubation. Each experiment was repeated three times and the data are represented as mean ± SE.

### Enzyme assay

The β-xylosidase activity was determined by a colorimetric technique based on determining the amount of the product p-nitrophenol (pNP) released from the substrate p-nitrophenyl-β-d-xylopyranoside (pNPX) (Katapodis et al. 2006). The reaction mixture (50 mM phosphate (pH 7.0), 5 mM pNPX, an appropriate amount enzyme, total volume 0.25 mL) was incubated for 10 min at 55°C, and then the reaction was terminated by adding 0.75 mL of 2 M Na_2_CO_3_. The pNP produced was quantified by measuring the absorbance at 410 nm. One unit (U) of β-xylosidase activity was defined as the amount of enzyme producing 1 μmol of pNP per minute.

### GenBank accession numbers

All the gene sequences used in the study are retrieved from the GenBank database, and their corresponding GenBank numbers were listed as follows: the *P. thermophila* J18 β-1,4-xylosidase gene *PtXyl43*, GU937001.1 (Teng et al. 2011); the codon-optimized PtXyl43 gene, MF818057; the *Citrobacter* sp. P (CGMCC No. 7536) chitinase gene *CHIX*, KC290945 (Xu et al. 2016); the hIL-2 gene *hIL*-*2*, AAA59140.1 (Robb 1984); the *N*-acyl homoserine lactonase (aiiO-AIO6) gene *aiiO*-*AIO6* from *Ochrobactrum* sp. M231, JN572045 (Zhang et al. 2011).

## Results

### X12345 secretion is a two-step process, and the second step is accompanied by periplasmic leakage

**Fig. 1:**
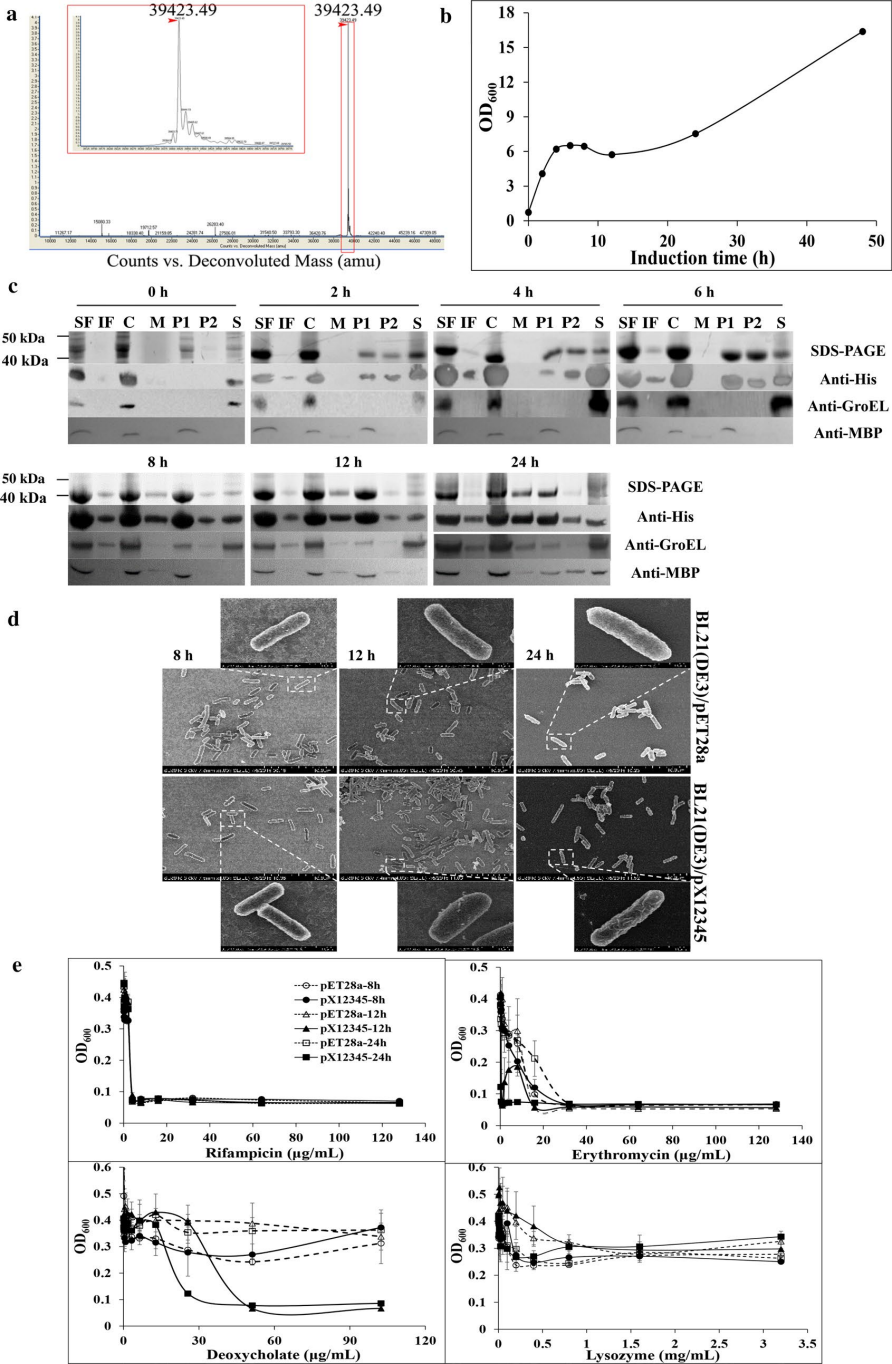
Growth, susceptibility and morphology of *E. coli* BL21(DE3)/pX12345 after lactose induction and subcellular location of X12345 in *E. coli* BL21(DE3). **a** Q-TOF MS of purified extracellular X12345 from BL21(DE3)/pX12345. The major peak has a molecular mass of 39,423.49 Da. **b** Growth curve of BL21(DE3)/pX12345 after lactose induction. The cellular density of bacterial cultures was determined at each indicated time points after induction with 2% lactose in TB medium by measuring optical densities at 600 nm. **c** Analysis of solubility and subcellular location of X12345 expressed in BL21(DE3). Cultures of BL21(DE3)/pX12345 at different induction time points were harvested and fractionated as described in “Materials and methods” section. Protein samples from these fractions were analyzed by 12% SDS-PAGE and western blotting with antibodies to his-tag of X12345, MBP, and GroEL. The lane loadings are directly comparable. *SF* soluble fraction, *IF* insoluble fraction, *C* cells, *M* medium, *P1* periplasm fraction (hypertonic solution fraction), *P2* periplasm fraction (hypotonic solution fraction), *S* spheroplasts. **d** FESEM of BL21(DE3)/pET28a and BL21(DE3)/pX12345 after lactose induction for each indicated time. **e** Susceptibility of induced BL21(DE3)/pX12345 or BL21(DE3)/pET28a to antibiotics and other compounds. After lactose induction for 8 (circles), 12 (triangles) or 24 h (squares), BL21(DE3)/pX12345 (solid line) or BL21(DE3)/pET28a (break line) cells were cultured aerobically at 37 °C in LB medium with different concentrations of erythromycin, rifampicin, deoxycholate or lysozyme for ~ 12 h, and cell growth was measured at OD_600_. Results are mean ± SD of triplicate observations

As previously reported, assessment by TatP 1.0 (Bendtsen et al. 2005) and SignalP 4.0 (Petersen et al. 2011) indicated that PtXyl43 (also named as X12345 herein) does not encode a twin-arginine signal peptide or non-twin-arginine signal peptide from bacteria or eukaryote (Teng et al. 2011). The Q-TOF MS of the purified extracellular X12345 from *E. coli* BL21(DE3)/pX12345 contained only one major peak (*m/z* = 39,423.49; Fig. 1a), which was consistent with the calculated molecular mass for X12345 with N-terminal methionine excision and C-terminal (His)_6_ tag (39,422.8 Da), confirming that no signal peptide is present in X12345.

*Escherichia coli* BL21(DE3)/pX12345 cell growth after induction was monitored by measuring absorbance at 600 nm. The recombinant cells were induced with 2% lactose at early exponential growth phase (OD_600_ value of 0.6–0.8). After 4 h induction, recombinant cells enter into stationary phase in which the slightly reduced OD_600_ value was observed between 8 and 12 h after induction, which corresponded to the start time of secretion of X12345 to the culture medium (Fig. 1b, c). Then the OD_600_ value was constantly increased after 12 h induction (Fig. 1b). This indicated that slight cell lysis occurred during the secretion of X12345 to the culture medium. Probably periplasmic leakage caused by X12345 secretion (Fig. 1c) further leading to instability of the inner membrane and then resulting in cell lysis.

Cell morphology changes were examined by FESEM. As shown in Fig. 1d, in the early stage (after 8 h induction) of X12345 excretion from cell to the extracellular milieu, the morphology of recombinant cells was similar to that of the control cells. As the induction time increased, the recombinant cells after 12 h induction was more swelling and coarse than the control cells. This might be due to that the large increase of X12345 in the cytosol and periplasm stretched cells and its outer membrane. After 24 h induction, both recombinant cells and control cells showed corrugated surface, and they remained unaltered rod shape, but recombinant sample has higher ratio of surface-shrink cells. Presumably, swelling recombinant cells export large amounts of X12345 from the periplasmic space to the extracellular milieu, which leads to more surface shrink of recombinant cells compared with control cells.

The membrane sensitivity of cells expressing X12345 to agents including rifampicin, erythromycin, deoxycholate, and lysozyme, was tested. Both recombinant cells and control cells are sensitive to rifampicin at different induction time. In the early stage (after 8 h induction) of X12345 excretion to the extracellular milieu, recombinant cells shows similar membrane sensitivity to control cells (Fig. 1e). Accompanying the extension of the excretion time (after 12 or 24 h induction), compared to control cells, recombinant cells are more sensitive to erythromycin and deoxycholate, and have a slightly increased sensitivity to lysozyme (Fig. 1e). These results indicated that the excretion of X12345 increased the membrane sensitivity of recombinant cells.

To investigate if the secretion of X12345 is a result of cell lysis or periplasmic leakage, we determined the subcellular locations of X12345, cytoplasmic GroEL, and periplasmic MBP by western blotting (Fig. 1c). X12345 was observed to secrete gradually into periplasm after 2 h induction and to begin exporting to the culture medium after 8 h induction, while trace amount of GroEL and MBP were observed in periplasm and the culture medium until 8 h after induction, respectively. It suggested that the transport of X12345 from cytosol to periplasm was not caused by cell lysis, while the export of X12345 from periplasm to extracellular milieu was accompanied by slightly periplasmic leakage.

These above results showed that the periplasm transportation of β-1,4-xylosidase is not due to cell lysis while its extracellular export is accompanied by periplasmic leakage. Moreover, the excretion of X12345 changed cell morphology and increased membrane sensitivity. Taken together, X12345 secretion may be the result of semi-specific secretion.

### X234 is the conformational determinant necessary for X12345 secretion

**Fig. 2:**
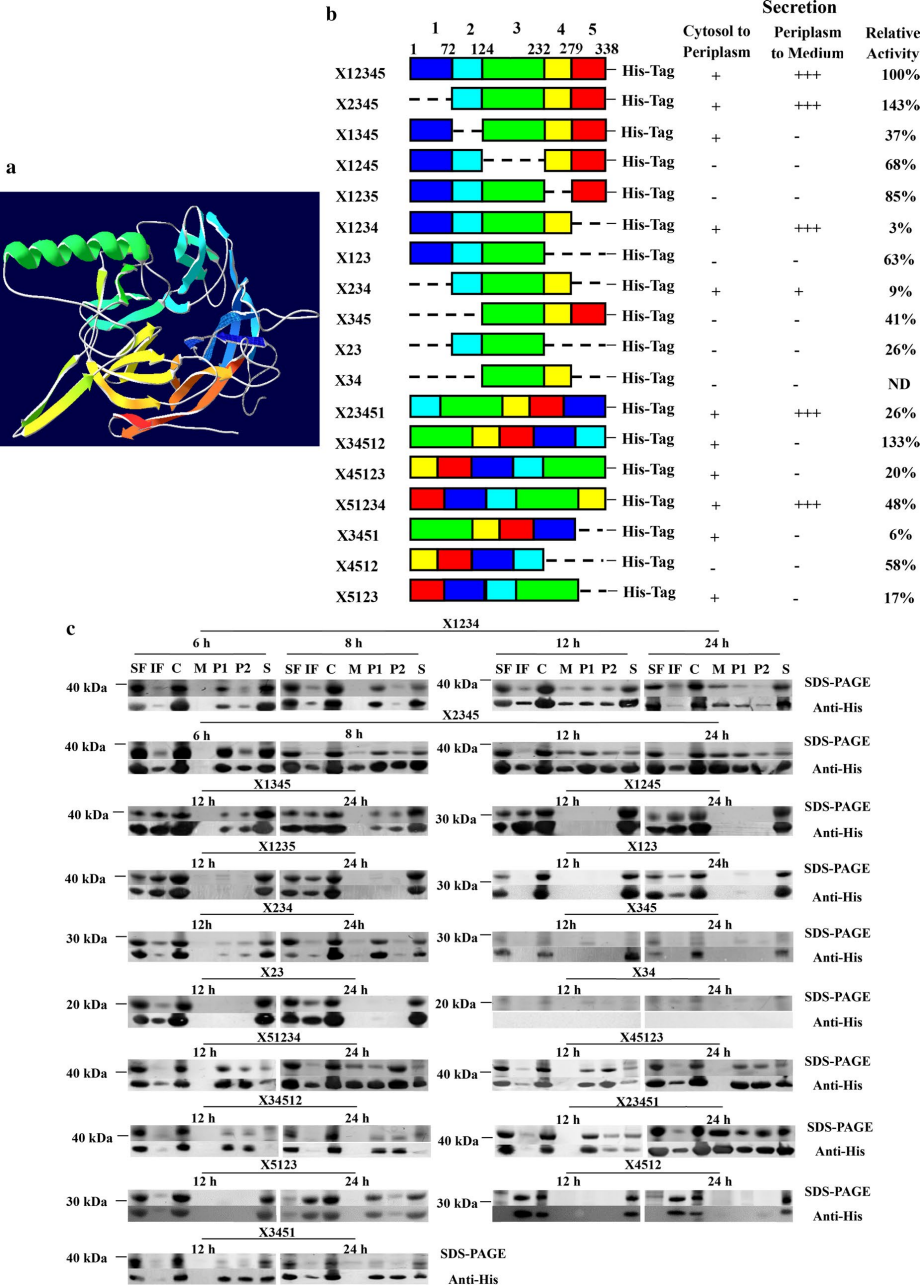
Construction of X12345 mutants and their secretion in *E. coli* BL21(DE3). **a** Predicted structure of X12345. The 3D structure of X12345 was predicted by Swiss-Model using the template arabinan-specific α-1, 2-arabinofuranosidase from *Cellvibrio japonicas* (PDB code, 3QED; 2.99-Å resolution), and colored from the N-terminus (blue) to the C-terminus (red). **b** Blade organization of the full-length X12345 and its mutants and their secretion in BL21(DE3). The regions of the 1 (blue), 2 (aqua), 3 (green), 4 (yellow) and 5 (red) blades were determined according to the predicted X12345 structure. Each gene was cloned into pET-28 (**a**) and expressed in BL21(DE3). +, +++ and −, secretion, high-level secretion and no secretion of target protein by BL21(DE3) after 24-h induction, respectively. The relative activity of purified mutants were also shown. ND, not detected. **c** Subcellular location of mutants of X12345 in BL21(DE3) after lactose induction for different time periods. The lane loadings are directly comparable. *SF* soluble fraction, *IF* insoluble fraction, *C* cells, *M* medium, *P1* periplasm fraction (hypertonic solution fraction), *P2* periplasm fraction (hypotonic solution fraction), *S* spheroplasts

Aided by SWISS-MODEL (Bordoli et al. 2009), the GH family 43, arabinan-specific α-1,2-arabinofuranosidase from *Cellvibrio japonicus* was used as the template [PDB code, 3qedD; 2.99-Å resolution; with 37.96% sequence identity to X12345; (Cartmell et al. 2011)] to predict the structure of X12345. The result showed that X12345 has a typical five-bladed β-propeller structure (Fig. 2a), common to all GH family 43 members (Brux et al. 2006). This β-propeller structure contains five radially oriented blades (labeled 1–5 in Fig. 2a) separated by ~ 72° with blades 2-4 forming an arch and blades 1 and 5 located next to each other (Brux et al. 2006).

To delineate the structure-secretion relationship for X12345, blade-deletion mutants of X12345 were generated, expressed in *E. coli*, and tested for secretion (Fig. 2b). Their expression levels and intra- and extracellular locations were characterized by SDS-PAGE and western blotting (Fig. 2c). All mutants except X34 and X4512 were expressed as both soluble and insoluble forms (Fig. 2c), which excluded that protein’s insolubility is a primary cause of the poorly secretion of these mutants. The majority of mutants, except X2345 and X34512, showed decreased activity toward pNPX compared with the wild-type enzyme X12345. These secretable mutants, such as X1234, X2345, X234, X51234 and X23451, showed activity equaling to 3 to 143% the activity of X12345 (Fig. 2b). This suggested that the secretion is irrelevant to the catalytic activity.

Among single-blade deletion mutants (e.g., X2345, X1345, X1245, X1235 and X1234), when blade 2, 3, or 4 was deleted, the expression levels were normal but secretion was adversely affected (Fig. 2c). X1245 and X1235 can not be secreted to either periplasm or the medium. X1345 can be translocated to periplasm but not to extracellular milieu. However, no effect on the secretion level was observed when blades 1 or 5 was deleted (Fig. 2b, c). These results showed that the three innermost blades are important for secretion. We next asked if the three blades were sufficient for secretion. The blades 1 and 5-deleted mutant X234 and other two mutants (X123 and X345) were constructed and expressed. For X123 and X345, the translocation from cytosol to periplasm was blocked. X234 can be transferred to periplasm and exported to the medium. However the excretion levels were significantly lower than those of X12345, X1234 or X2345. To test whether smaller secretion determinant exists, we further truncated X234 as X23 and X34. X23 can not translocate from cytosol to periplasm, and X34 is expressed at a very low level that is undetectable by western blotting (Fig. 2c). Mutants X1345 and X34, without the intact blades 234, were abolished in the periplasmic and extracellular secretion, and blades 1 or 5 significantly increased the extracellular secretion of X234. These results suggested that X234 plays a key role for periplasmic secretion, while blades 1 or 5 facilitates the extracellular secretion of X234.

We next investigated whether the circular structure (X234) was sufficient for secretion. The circular mutants (X51234, X45123, X34512, X23451) were constructed and expressed (Fig. 2c). Like X12345, X51234 and X23451 showed normal secretion level. However, X34512 and X45123, where blades of 2 and 3 or 3 and 4 were separated, were blocked in periplasm and could not be translocated from periplasm to the medium. The circular mutants were further truncated one blade from the N- or C-terminus to generate new blade deletion mutants (X5123, X4512 and X3451). These three mutants could not be exported to extracellular medium. These results further confirms that noncovalent linkage of blades 2, 3 and 4 can transport mutants to periplasm, while covalent and sequential binding of blades 2, 3 and 4 are essential for the excretion of mutants.

### Secretion of proteins fused to X12345

**Fig. 3:**
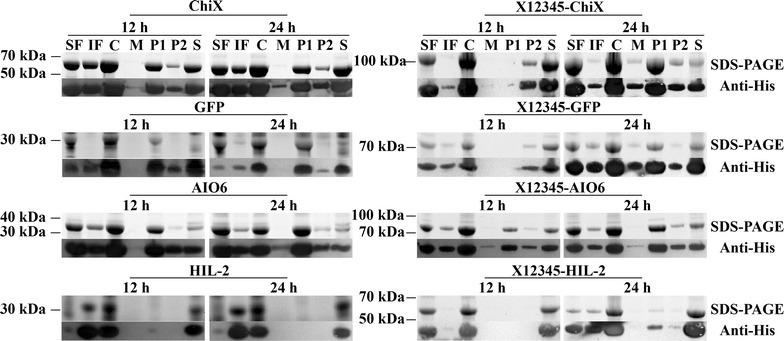
Subcellular locations of the X12345–ChiX, GFP, AIO6, hIL-2. Each of the for ‘passenger’ proteins, AIO6, hIL-2, and GFP, were expressed in *E. coli* BL21(DE3) harboring pET28a(+) or pX12345LT. The lane loadings are directly comparable. *SF* soluble fraction, *IF* insoluble fraction, *C* cells, *M* medium, *P1* periplasm fraction (hypertonic solution fraction), *P2* periplasm fraction (hypotonic solution fraction), *S* spheroplasts

Considering similar excretion ability among X12345, X1234 and X2345 (Fig. 3), X12345 was chosen to test its capacity as a carrier. We individually fused green fluorescent protein [GFP, a 27-kDa hydrophilic reporter protein; (Cormack et al. 1996; Yang et al. 1996)], human interleukin-2 [hIL-2, a 15-kDa hydrophobic protein; (Robb 1984)], the *N*-acyl homoserine lactonase [AIO6, 29 kDa; (Zhang et al. 2011)], the secretable signal peptide-lacking chitinase from *Citrobacter* sp. P (ChiX, 54 kDa); (Xu et al. 2016) to the C terminus of X12345. The locations of each of these proteins were characterized after lactose induction for 12 and 24 h. As shown in Fig. 3, similar to X12345 and ChiX, X12345–ChiX can be secreted into extracellular milieu; X12345–GFP showed extracellular secretion, while GFP were only secreted to periplasm. X12345–hIL-2 was more soluble than hIL-2, and can be secreted into periplasm, while hIL-2 were blocked in cytoplasm. Like AIO6, X12345–AIO6 can only be secreted to periplasm. These results suggested that X12345 can improve the periplasmic or extracellular secretion of proteins.

## Discussion

Since the mechanisms for non-classical secretion remains controversial and appear to be even more complex. The identification of the exact excretion mechanism has been the primary task. There have been few reported cases of excretion of signal peptide-lacking proteins in laboratory stain *E. coli* BL21(DE3). *Thermobifida fusca* signal peptide-lacking cutinase was expressed extracellularly in *E. coli* BL21(DE3). Cell leakage caused by the limited phospholipid hydrolysis of cutinase was proposed to explain the potential mechanism for the cutinase extracellular release (Su et al. 2013). In this study, extracellular secretion of X12345 was found to be independent of its activity because secretable X1234 and X234 were almost inactive. We confirm the hyper-secretion of signal-lacking β-1,4-xylosidase in *E. coli* to be a two-step process: the first step to periplasm is not due to cell lysis but the second step to extracellular milieu is accompanied by periplasmic leakage. This may be because the X12345 secretion to the periplasm caused instability of the outer membrane as described in semi-specific secretion (Georgiou and Segatori 2005). Our results also support the opinion that extracellular secretion of signal peptide-lacking proteins is associated with periplasmic leakage (Wang et al. 2013; Gotz et al. 2015; Ebner et al. 2016).

For this report, the low extracellular secretion level of the smallest secretable mutant X234 indicates that covalent linkage of central blades 2–4 is necessary, but not sufficient for the hyper-excretion. N-terminal blade 1 or C-terminal blade 5 appears to be needed to maintain the intact excretory structure associated with blades 2–4. According to the X12345 structural model, its tertiary structure appears to be circular: blades 2–4 form an arch which acts as the conformational determinant(s) necessary for secretion, and this arch may be maintained by blade 1 and 5. Because removal of only blade 1 or 5 had no apparent effect on secretion of X2345 and X1234, inclusion of either blade 1 or 5 may be sufficient to maintain the conformation of blades 2–4. With the simultaneous removal of blades 1 and 5, the interactions necessary to maintain the conformations of blades 2–4 are probably unstable, which would severely reduce excretion.

Recently, non-classical secretable proteins have been reported as signals for exporting recombinant proteins to the culture medium of *Bacillus subtilis* (Wang et al. 2015; Chen et al. 2016), it is important to develop more carrier proteins for recombinant proteins secretion through non-classical pathway. The size, the native localization and gene source of reporter proteins do not seem to affect their secretion, and the recombinant target protein itself plays an important role in protein secretion when using the non-classical secretory pathway (Wang et al. 2015; Chen et al. 2016). Similar results were found in this study. Although X12345 can be excreted at unusual high level, only the excretable ChiX (with large size, 54 kDa) and periplasmic GFP (with small size, 27-kDa) can be exported to extracellular space by X12345, while AIO6 and hIL-2 can only be transported to periplasm. We speculate that ChiX and GFP contain conformational motifs that help to maintain the secretion conformation of fusion proteins better than AIO6 and hIL-2. These data indicate that X12345 has the ability to carry proteins from *E. coli* cells into medium, especially for proteins that are compatible with X12345 in terms of secretion.

In summary, this study provides new insights into secretory mechanism of secretable signal peptide-lacking proteins in *E. coli*. To our knowledge, this is the first to definitively identify the conformational determinants for secretion of a signal peptide-lacking GH family 43 β-xylosidase. This finding also has application potential for the secretion production of recombinant proteins. In the future, we intend to explore how X12345 is recognized and translocated across cell inner membrane, and how X12345 and cell outer membrane interact to achieve excretion. Furthermore, an X12345 three-dimensional structure will be obtained and analyzed to study why blades 2–4 contain the conformational information necessary for secretion.

## Additional file

**Additional file 1.** Additional tables.

## Abbreviations

GH43: glycoside hydrolase family 43
T2SS: type II secretion system
T3SS: type III secretion system
TCP: toxin-coregulated pilus
LB: Luria–Bertani
TB: Terrific Broth
EDTA: ethylene diamine tetraacetic acid
DNA: deoxyribonucleic acid
PCR: polymerase chain reaction
SDS-PAGE: sodium dodecylsulphate polyacrylamide gel electrophoresis
PBS: phosphate buffer solution
MS: mass spectrometry
Q-TOF: quadrupole time-of-flight
FESEM: field emission scanning electron microscopy
CFU: colony forming units
pNP: p-nitrophenol
pNPX: p-nitrophenyl-β-d-xylopyranoside
U: unit
