# Supplementary material for: Conformational determinants necessary for secretion of Paecilomyces thermophila β-1,4-xylosidase that lacks a signal peptide
Source: AMB Express. 2018 Jan 24;8:11. doi: 10.1186/s13568-018-0542-2 (PMC5783984; doi:10.1186/s13568-018-0542-2)
Supplement: Supplementary file 1 — Additional file 1. Additional tables. [file 13568_2018_542_MOESM1_ESM.docx]

**Additional Information**

**Additional Tables**

**Table S1 Bacterial strains and plasmids used in this study**

| **Strain/Plasmid** | **Description** | **Source** |
| --- | --- | --- |
| *E. coli* Trans1-T1 | *F ^–^*φ80(*lac*Z)ΔM15Δ*lac*X74*hsd*R(rk*^–^*, mk^+^)Δ*rec*A1398*end*A1*ton*A | Transgen |
| *E. coli* BL21 (DE3) | *F ^–^ompThsdS_B_*(*r_B_^–^m_B_^–^*) *gal dcm*(DE3) | Novagen |
| pET28a | 5.4 kb, f1 ori, T7 promoter, Kan^R^ | Novagen |
| pX12345 | the full-length codon-optimized *PtXyl43* gene in *Xba*I-*Xho*I site of pET28a | This study |
| pX2345 | a truncated *PtXyl43* gene (deletion of blade 1) in *Nco*I-*Xho*I site of pET28a | This study |
| pX1345 | a truncated *PtXyl43* gene (deletion of blade 2) in *Xba*I-*Xho*I site of pET28a | This study |
| pX1245 | a truncated *PtXyl43* gene (deletion of blade 3) in *Xba*I-*Xho*I site of pET28a | This study |
| pX1235 | a truncated *PtXyl43* gene (deletion of blade 4) in *Xba*I-*Xho*I site of pET28a | This study |
| pX1234 | a truncated *PtXyl43* gene (deletion of blade 5) in *Xba*I-*Xho*I site of pET28a | This study |
| pX123 | a truncated *PtXyl43* gene (deletion ofblade 4 and 5) in *Xba*I-*Xho*I site of pET28a | This study |
| pX234 | a truncated *PtXyl43* gene (deletion of blades 1 and 5) in *Nco*I-*Xho*I site of pET28a | This study |
| pX345 | a truncated *PtXyl43* gene (deletion of blades 1 and 2) in *Nco*I-*Xho*I site of pET28a | This study |
| pX23 | a truncated *PtXyl43* gene (deletion of blades 1, 4 and 5) in *Nco*I-*Xho*I site of pET28a | This study |
| pX34 | a truncated *PtXyl43* gene (deletion of blades 1, 2 and 5) in *Nco*I-*Xho*I site of pET28a | This study |
| pX24 | a truncated *PtXyl43* gene (deletion of blades 1, 3 and 5) in *Nco*I-*Xho*I site of pET28a | This study |
| pX23451 | a circularly *PtXyl43* mutantgene (fused blade 1 to blade 5 for separating blades of 1 and 2) in *Nco*I-*Xho*I site of pET28a | This study |
| pX34512 | a circularly *PtXyl43* mutantgene (fused blades 1 and 2 to blade 5 for separating blades of 2 and 3) in *Nco*I-*Xho*I site of pET28a | This study |
| pX45123 | a circularly *PtXyl43* mutantgene (fused blades 1, 2 and 3 to blade 5 for separating blades of 3 and 4) in *Nco*I-*Xho*I site of pET28a | This study |
| pX51234 | a circularly *PtXyl43* mutantgene (fused blades 1, 2 ,3 and 4 to blade 5 for separating blades of 4 and 5 ) in *Nco*I-*Xho*I site of pET28a | This study |
| pX3451 | a truncated *X23451* gene (deletion of blade 2) in *Nco*I-*Xho*I site of pET28a | This study |
| pX4512 | a truncated *X34512* gene (deletion of blade 3) in *Nco*I-*Xho*I site of pET28a | This study |
| pX5123 | a truncated *X45123* gene (deletion of blade 4) in *Nco*I-*Xho*I site of pET28a | This study |
| pX12345LT | Fusion gene, including *PtXyl43 gene*, sequence encoding the flexible (GGGGS)_3_ linker and the TEV-protease cleavage site (ENLYFQG), in *Nco*I-*Eco*RI site of pET28a | This study |
| pX12345-GFP | the GFP gene in *Eco*RI-*Xho*I site of pX12345LT | This study |
| pX12345-AIO6 | the AIO6 gene in *Eco*RI-*Xho*I site of pX12345LT | This study |
| pX12345-HIL | the HIL gene in *Eco*RI-*Xho*I site of pX12345LT | This study |
| pX12345-ChiX | the ChiX gene in *Eco*RI-*Xho*I site of pX12345LT | This study |

**Table S2 Primers used in this study**

| **Primers** | **Sequence** |
| --- | --- |
| X12345-forward | CAATTCCCCTCTAGAAATAATTTTGTTTAACTTTAAGAAGGAGATATACCATGAGCAAC |
| X12345-reverse | TGGTGGTGCTCGAGATGCTGCGGTTCTTTCAGGCGAATTTTG |
| X1234-reverse | TGGTGGTGCTCGAGAATGCGGCCCTGATAAATAAACGGGCCAAA |
| X123-reverse | TGGTGGTGCTCGAGATGATCATCCGCCAGAATCGGCTGGCGGGTTT |
| X2345-forward | AGATATACCATGGGCGATGATGTGCCGTGGGTGAGCAAACAGCTGT |
| X345-forward | AGATATACCATGGGCCCGGAACCGATTCCGGGCAGCTATAGCATTGAT |
| X24-fusion reverse | ACCACGCCGCTTCAAAAAAGCGGCGATCTTCCGGTTTAAACGGGCCATGCGGTT |
| X24-fusion forward | AACCGCATGGCCCGTTTAAACCGGAAGATCGCCGCTTTTTTGAAGCGGCGTGGT |
| X13-fusion reverse | TGCTATAGCTGCCCGGAATCGGTTCCGGCGCATGCAGCGCCACGCCATGATCGGT |
| X13-fusion forward | ACCGATCATGGCGTGGCGCTGCATGCGCCGGAACCGATTCCGGGCAGCTATAGCA |
| X35-fusion reverse | TGGTCCAGCCCACCACCGGTTCCAGATGATCATCCGCCAGAATCGGCTGG |
| X35-fusion forward | CCAGCCGATTCTGGCGGATGATCATCTGGAACCGGTGGTGGGCTGGACCA |
| X23451-forward | AGATATACCATGGGCGATGATGTGCCGTGGGTGAGCAAACAGCT |
| X51-fusion reverse 1 | ATATGGGTCACCAGCGGTTTCGGGTTGCTATGCTGCGGTTCTTTCAGGCGAATTTT |
| X51-fusion forward | AAAATTCGCCTGAAAGAACCGCAGCATAGCAACCCGAAACCGCTGGTGACCCATAT |
| X23451-reverse | TGGTGGTGCTCGAGCGCATGCAGCGCCACGCCATGATCGGTCA |
| X34512-forward | AGATATACCATGGGCCCGGAACCGATTCCGGGCAGCTATAGCAT |
| X34512-reverse | TGGTGGTGCTCGAGTTCCGGTTTAAACGGGCCATGCGGTTCAT |
| X45123-forward | ATACCATGGGCGATCGCCGCTTTTTTGAAGC |
| X51-fusion reverse 2 | ATATGGGTCACCAGCGGTTTCGGGTTGCTATGCT |
| X45123-reverse | GGTGCTCGAGATGATCATCCGCCAGAATC |
| X51234-forward | AGATATACCATGGGCCTGGAACCGGTGGTGGGCTGGACCACCCAT |
| X51234-reverse | TGGTGGTGCTCGAGAATGCGGCCCTGATAAATAAACGGGCCAAA |
| X12345-linker-forward | CATGCCATGGGCAGCAACCCGAAACCGCTGGTGACCCATATTTATACCGCGGATCCGA |
| X12345-linker-reverse | GCTCGAATTCGCCCTGAAAATACAGGTTTTCGCTACCACCACCGCCGCTGCCACCGCCA |
| X2345-linker-forward | CATGCCATGGGCGATGATGTGCCGTGGGTGAGCAAACAGCTGTGGGCGCCGGAT |
| HIL2-forward | CCGGAATTCGcgccgaccagcagcagcaccaaaaaaacccagct |
| HIL2-reverse | TTTTCCTTTTGCGGCCGCGGTCAGGGTGCTAATAATGCTCTGGCAAAAGGTAAT |
| GFP-forward | CCGGAATTCGTGAGCAAGGGCGAGGAGCTGTTCACCGGGGTGGTGCCCAT |
| GFP-reverse | TTTTCCTTTTGCGGCCGCCTTGTACAGCTCGTCCATGCCGAGAGTGATCC |
| AIO6-forward | CCGGAATTCAAATCCCATGAAATCGAGACCAGTCACGGTCGCAT |
| AIO6-reverse | TTTTCCTTTTGCGGCCGCGGCCGTGCAGTCGCGCATGAAACGCTGCAGATAAT |
| ChiX-forward | CCGGAATTCACTAACAGCAAACTGGTACAAAATGATTCGCTCAGTACA |
| ChiX-reverse | TTTTCCTTTTGCGGCCGCTGCCTTCGTAATACCTTTAAAATAGAACGGTCTCATAT |
